# Insights from deep mutational scanning in the context of an emerging pathogen

**DOI:** 10.1042/BST20253033

**Published:** 2025-09-04

**Authors:** Melissa J. Call, Matthew E. Call, Xinyu Wu

**Affiliations:** 1 The Walter and Eliza Hall Institute of Medical Research, Parkville, VIC, Australia; 2 Department of Medical Biology, University of Melbourne, Parkville, VIC, Australia

**Keywords:** deep mutational scanning, multiplexed assays of variant effects, nucleocapsid, SARS-CoV-2, spike, viral proteases

## Abstract

Deep mutational scanning (DMS), a high-throughput method leveraging next-generation sequencing, has been crucial in mapping the functional landscapes of key severe acquired respiratory syndrome-coronavirus 2 (SARS-CoV-2) proteins. By systematically assessing thousands of amino acid changes, DMS provides a framework to understand Angiotensin-converting enzyme 2 (ACE2) binding and immune evasion by the spike protein, mechanisms and drug escape potential of the main and papain-like viral proteases and has highlighted areas of concern in the nucleocapsid protein that may affect most currently available rapid antigen testing kits. Each application has required the design of bespoke assays in eukaryotic (yeast and mammalian) cell models, providing an exemplar for the application of this technique to future pandemics. This minireview examines how DMS has predicted key evolutionary changes in SARS-CoV-2 and affected our understanding of SARS-CoV-2 biology, specifically highlighting their relevance for therapeutics development.

## Introduction

**Figure 1:**
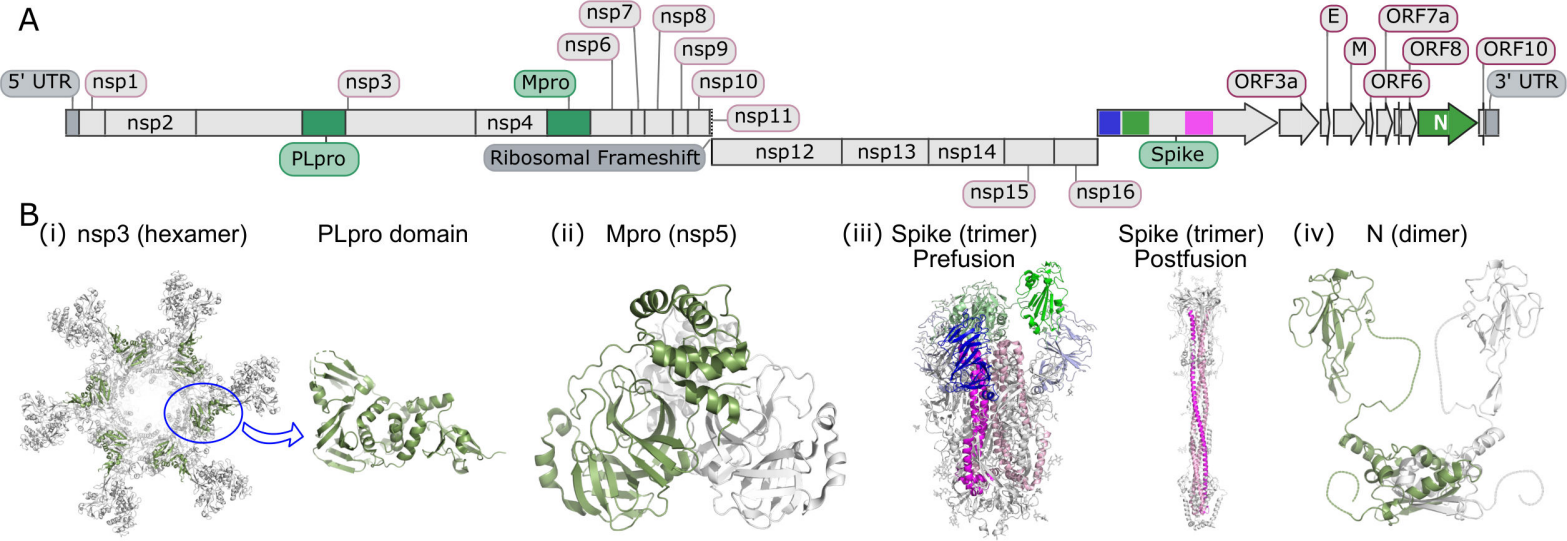
The SARS-CoV-2 genome and proteins subject to DMS. (**A**) The SARS-CoV-2 genome to scale. The regions encoding mature proteins are labelled. Various DMS assays have been performed on the complete sequences of Mpro and nucleocapsid protein, as well as the PLpro domain of nsp3 (labelled and highlighted in green). The spike protein has complete DMS data for the N-terminal domain (dark blue), RBD (green) and partial DMS data for the S2 domain (pink). Grey regions have no available DMS data. (**B**) Structures of the proteins subject to DMS. (i)The position of the PLpro domain in nsp3 [8YAX] [2] is circled and expanded. (ii) Mpro is an obligate dimer with one chain shown in green and the other in grey [6Y2E] [3]. (iii) Spike protein is shown with colouring matching a) in its prefusion form with 1 RBD (green) in the up position [6VSB] [4]. The NTD (blue) and RBD domains detach from the S2 domain (pink) in the postfusion form of the spike [8FDW] [5]. (iv) The two folded domains of N are shown (residues 48–161 & 238–366) based on an Alphafold3 [6] model. Additional sections are unstructured and linked with dotted random coil. E, envelope; M, membrane; Mpro, main protease; N, nucleocapsid; nsp, non-structural protein; PLpro, papain-like protease.

The coronavirus disease 2019 (COVID-19) pandemic emerged at a time when recent scientific advances could be rapidly deployed to address the growing threat. Technologies such as next-generation DNA sequencing allowed rapid characterisation of the severe acquired respiratory syndrome-coronavirus 2 (SARS-CoV-2) genome [1], which is organised as a single-stranded positive-sense RNA molecule approximately 30 kilobases in length that encodes 29 proteins. Sixteen of these are non-structural proteins (nsps), initially translated as polyproteins open reading frame (ORF) 1a and 1ab, that require additional processing by proteases for maturation (Figure 1a). Sequencing of viruses from successive waves of infection afforded an unprecedented readout of viral evolution, showing the changes SARS-CoV-2 used to adapt to human host proteins and evade immunity [7]. Cryo-electron microscopy gave us the first atomic resolution images of the SARS-CoV-2 spike protein within weeks [8,9], and encapsulated mRNA vaccine technology was leveraged to develop vaccines in record time and mitigate the threat to human health [10]. Deep mutational scanning (DMS) [11–13], a relatively new method that takes advantage of next-generation sequencing technology, was rapidly deployed to map the functional landscapes of four key SARS-CoV-2 proteins: the spike protein [8,9], main protease (Mpro) [14,15], papain-like protease (PLpro) [16] and the nucleocapsid (N) protein [17]. This approach enabled the systematic assessment of how individual amino acid changes influenced ACE2 binding, antibody escape, enzymatic activity and the ability to evade detection by rapid antigen testing kits. These studies revealed critical residues for viral infectivity and immune evasion and informed the design of vaccines, therapeutic antibodies, antiviral inhibitors and surveillance tools.

**Figure 2:**
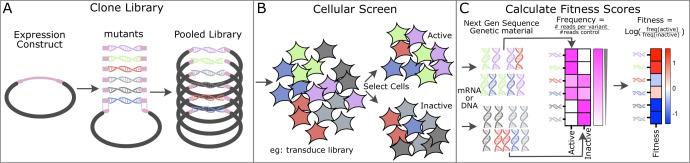
Overview of deep mutational scanning workflow. (**A**) A pooled library containing many variants of a protein of interest is cloned into an expression vector compatible with the downstream cellular system used to assess variant function. Often, primers or DNA fragments containing degenerate codons are cloned in a Gibson-style assembly to generate the pooled variant library. (**B**) The variant library is introduced into cells through viral transduction, transformation or transfection. Once installed into cells, selection is performed to separate cells based on the protein of interest’s function or abundance. (**C**) DNA or mRNA is recovered from selected populations and prepared for next-generation sequencing to enumerate variants. Read counts are converted into frequencies, and then a log ratio is calculated to define a fitness score.

DMS is a form of a pooled multiplexed assay where the structural and functional effects of protein variants are systematically assessed in a single experiment [11] (Figure 2). DMS screens must couple protein function to the genetic material used to produce the protein variant, which is often (but not always) accomplished by utilisation of a cellular system where the cell membrane provides a container to link protein and genetic material. Both eukaryotic and prokaryotic systems can be used for this purpose, with considerations around ease of use and the ability of the cellular system to support the protein of interest’s folding, function and post-translational modification driving the choice.

Cells are enriched or depleted based on the phenotype of the protein variant, which typically provides a survival or growth advantage or a change in a fluorescence-based readout. The frequency of protein variants in selected and non-selected populations is determined using read counts from next-generation sequencing of genetic material recovered pre- and post-selection. Here, we delve into the use of DMS during the COVID-19 pandemic, highlighting the different screening modalities used to uncover SARS-CoV-2 biology and the findings that influenced the pandemic response.

## Spike

The spike is a large (1273 amino acid) protein that forms a trimeric structure on the surface of the SARS-CoV-2 virus (Figure 1b(**iii**)). The receptor-binding domain (RBD) within the spike protein consists of approximately 200 amino acids and directly interacts with ACE2, initiating host cell invasion. Blocking the spike RBD-ACE2 interaction, generally by anti-RBD antibodies, results in sterilising immunity and halts infection.

### The receptor-binding domain

**Figure 3:**
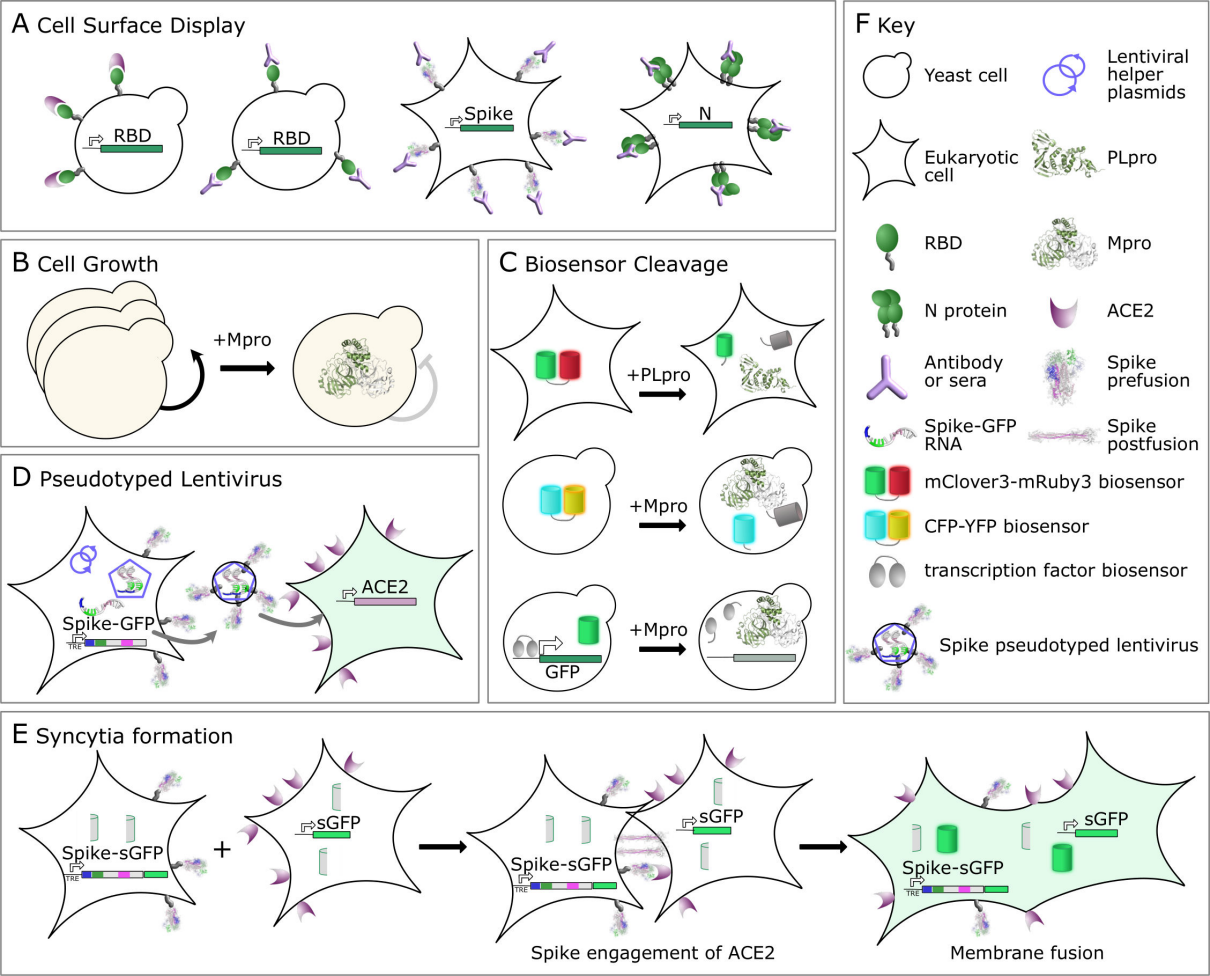
Overview of different assays performed to measure the sequence–function relationship of SARS-CoV-2 proteins. (**A**) Cell surface display. RBD on yeast measuring ACE2 and antibody binding. Full-length spike and N on the surface of eukaryotic cells. (**B**) Cell growth based on the Mpro retarding cell division through the cleavage of essential proteins. (**C**) Biosensor cleavage. Biosensors linked by PLpro or Mpro target sequences can be cleaved to decrease energy transfer between Förster resonance energy transfer (FRET) pairs or to break a transcription factor (DNA-binding domain-activation domain fusion) driving green fluorescent protein (GFP) expression. (**D**) Pseudotyped Lentivirus produced by installing spike-GFP into the genome of a cell. Upon transfection of lentiviral helper cells and doxycycline treatment, spike-GFP is transcribed into RNA and spike protein produced that can be packaged and collected as secreted pseudotyped virus. Pseudotyped virus is then used to infect an ACE2 expressing cell line that becomes GFP positive upon successful transduction. (**E**) Syncytia formation. Cells expressing full-length spike and a split GFP (sGFP) fragment are mixed with ACE2+ cells expressing the complementary GFP fragment. Syncytia formation, mediated by spike engagement with ACE2, is determined by the appearance of GFP+ cells. (**F**) Key for Figure. CFP, cyan fluorescent protein; Mpro, main protease; N, nucleocapsid; nsp, non-structural protein; PLpro, papain-like protease; RBD, receptor-binding domain; YFP, yellow fluorescent protein.

It is not surprising that the first deep mutational scans were performed on the RBD of the spike protein, with the first paper deposited in bioRXiv on 17 June 2020, and later published in *Cell* [8]. Using a yeast surface display system and screening more than 100,000 variants with 95.7% coverage of single-site substitutions (Figure 3a), the Bloom lab reported the sequence constraints on RBD folding and ACE2 binding. Flow cytometry was used to sort yeast cells with surface-displayed RBD stained with an antibody to an incorporated tag into four bins. Next-generation sequencing was then used to quantify the distribution of each variant among bins, which was converted into a mean fluorescence intensity score, with higher scores indicating proper folding and expression. Using fluorescently labelled recombinant ACE2 as the staining reagent, this same strategy can be used to construct titration curves and infer the relative ACE2 binding affinity of each variant in the library. While yeast display assays have been invaluable for these DMS studies, it’s worth noting that yeast glycosylation patterns differ from those in mammalian cells, potentially affecting the observed RBD–ACE2 interactions. Nevertheless, these foundational datasets map the structural landscape of the RBD, highlighting regions where mutations can enhance ACE2 binding or facilitate immune evasion and identifying constrained sites that the virus is less able to alter. The latter may be particularly valuable targets for the development of monoclonal antibodies (mAbs) that recognise epitopes that are conserved across SARS-CoV-2 clades.

Surveillance of SARS-CoV-2 variants has been a critical component in managing the COVID-19 pandemic, and the data obtained from DMS have both predicted viral evolution and provided a framework to understand the mechanisms underlying the increasing prevalence of certain variants [8,9,18–21]. In these early studies, spike N501Y, Q498H and Y453F were identified as among the strongest enhancers of ACE2 binding and were accessible to the virus with single base-pair mutations [8]. N501Y was indeed rapidly acquired, first observed in September 2020 in the Alpha variant (B.1.1.7), and it remains a defining feature in currently circulating variants [22]. Y453F was first identified in April 2020 and contributed to a significant wave (B.1.1.298) in Denmark towards the end of that year, involving mink to human transmission [23]. After this wave was controlled, the Y453F mutation was rarely observed. Q498H is not tolerated in combination with N501Y because a structural clash is introduced when both mutations are present [24]. Instead, Q498R is a common feature of currently circulating variants, likely synergising with N501Y to increase ACE2 binding. This highlights a limitation of DMS in that it typically assesses the function of single-site substitutions, while viral evolution is characterised by the accumulation of multiple mutations creating epistatic effects that may not be apparent from the datasets using the original Wuhan base sequence. Indeed, the potential for different evolutionary trajectories could be measured when comparing libraries created from Alpha, Beta, Delta, Eta and Omicron RBDs, providing one way in which this limitation can be circumvented using libraries with manageable levels of combinatorial diversity and arguing for ongoing use of DMS studies during an unfolding pandemic to inform variant surveillance efforts [25,26].

Early in the pandemic, neutralising mAbs were isolated from convalescent patients and combined into cocktails that could be used to treat naive infected individuals at risk of severe disease. The existing spike variant libraries were leveraged to epitope map nine human mAbs, determining all mutations that reduced binding and then mapping those mutations back to the surface of the RBD using the yeast surface display described above [27]. Most mutations cluster on the surface, indicating the antibody-RBD binding interface, but some mutations were found in isolated positions and have been proposed to act allosterically. These studies defined different binding classes, informing cocktail design to ensure that each class of neutralising antibody was included in the mixture to provide comprehensive protection and limit opportunities for the virus to accumulate mutations to evade all constituent mAbs.

While it was clear that RBD libraries could report on escape mutants to individual mAbs, it was surprising that insights could also be gleaned from screening against sera from convalescent individuals, where one might assume that the breadth of antibodies arising through natural immunity would mask any one escape mutant. Instead, when the neutralising activity of sera from 17 SARS-CoV-2 convalescent patients was measured, dominant epitope classes that were affected by single mutations were observed in the majority of subjects [28]. These studies identified E484 and structurally adjacent regions as being critical to population immunity, as well as a loop directly involved in ACE2 binding that encompasses residues 443–450. Indeed, at the time this study was published, E484K was already circulating in a subset of Alpha and Delta strains that were largely eliminated from circulation by the end of 2022 [22]. In early 2022, E484A became dominant as Omicron variants spread. The JN.1 variant emerged at the end of 2023, carrying the E484K mutation, perhaps as Alpha- and Delta-generated immunity waned. Mutations at other sites identified in this study have emerged as recently as 2024, when N450D became dominant in circulating strains. As the pandemic progressed, repeating these screens on the background of newly observed RBD sequences mapped the immune response and the positions that evolved under immune pressure [29,30].

As vaccines became widely available, DMS was used to compare the neutralising activity of sera from mRNA-1273-vaccinated individuals with those who were naturally infected with the virus [31]. These studies showed that vaccination with spike resulted in broader epitope coverage than natural infection, such that no single mutation negated neutralising activity in samples collected 36 days post two-dose vaccination, likely due to focusing the immune response on a single, highly relevant target. The simple yeast display system used in these studies has thus proven a powerful assay to map mutations that improve ACE2 binding and facilitate antibody escape. This nicely demonstrates the effectiveness of vaccination and continues to be used in the development of biologics of increasing complexity [32].

### Full-length spike

Earlier studies focused on the RBD, but this comprises less than 20% of the much larger spike protein, which is arranged as a trimer and undergoes significant conformational changes during receptor engagement and M fusion. The S1 domain of the spike contains the N-terminal domain (NTD) and the RBD, both of which are subject to immune selective pressure [33]. The RBD binds directly to ACE2, but the role of the NTD is less clear and may involve binding sugars on the host cell surface and/or modulating conformational changes in the S2 domain after host cell engagement [34]. During cell entry, the spike protein is cleaved by host proteases, releasing the S1 domain and allowing a rearrangement in the M-proximal S2 domain from a prefusion to postfusion conformation (Figure 1b(**iii**)). Conservation of the S2 sequence among betacoronaviruses makes this an attractive avenue to explore in the development of broadly neutralising antibody-based therapies [35].

Current vaccines include mutations to proline at K986 and V987 that both increase spike expression levels and stabilise the prefusion form [36]. To determine additional mutations with these properties, the Wu laboratory developed M fusion assays that measured cell surface levels of the spike on mammalian cells by flow cytometry [37] (Figure 3a). M fusion was measured by combining cells that expressed both spike and a free split green fluorescent protein (GFP) with target cells expressing the complementary GFP domain (Figure 3e). When these assays were used in conjunction with DMS targeting a region of the S2 domain necessary for the formation of the postfusion conformation (with 99.9% coverage of possible single-site substitutions), additional residues that both increased expression and prevented M fusion were found. While K986P and V987P have proven sufficient in current vaccine formulations, the results reported in this work provide a framework to rapidly screen for desirable mutations in the entry receptors of viruses with future pandemic potential.

Each of the studies described above isolated certain functions of the spike, but none assessed the overall ability of the spike to mediate viral entry into the cell. To this end, the Bloom lab developed a pseudotyped lentiviral model system [19] (Figure 3d). This approach involves first transducing human embryonic kidney 293T (HEK293T) cells with a library of doxycycline-inducible variant spike open reading frames. When these cells are then transfected with packaging vectors and treated with doxycycline, each will simultaneously produce a variant spike protein and the lentiviral RNA encoding it, yielding secreted virions whose ability to orchestrate a second round of viral entry is directly linked to their spike genotype.

This elegant system has been used to understand the evolution of multiple emerging SARS-CoV-2 clades, where complete scans of the RBD were combined with sparse screening of the remaining spike protein that focused on the sites that varied among clades. The effects of mutations in the RBD were largely in line with results found in the yeast system, showing that mutations reducing ACE2 binding similarly reduced infectivity. Mutations in the spike also induced differences in susceptibility to antibody neutralisation via mechanisms that included modulating the equilibrium of the RBD between the open (ACE2-binding) and closed (non-binding) forms (Figure 1b(**iii**)), where the latter limits surface exposure to neutralising antibodies.

Now that SARS-CoV-2 is firmly established in the human population, the assays discussed above are increasingly being used to assess biologics that aim to limit SARS-CoV-2 mutations and provide broad protection from a disease that still remains a leading cause of death in vulnerable populations [32,38,39].

## SARS-CoV-2 proteases

Proteases are critical enzymes for many viruses, including retroviruses such as human immunodeficiency virus subtype 1 (HIV-1), flaviviruses such as hepatitis C virus (HCV), enteroviruses and coronaviruses, among others. This is because they play key roles in processing the viral proteins necessary for replication. HIV-1 protease inhibitors ritonavir and lopinavir have been successfully used in antiretroviral therapy, and HCV protease inhibitors boceprevir and telaprevir have significantly improved treatment outcomes for HCV [40]. With success in these viruses, the SARS-CoV-2 proteases were rapidly identified as potential therapeutic targets, with Mpro being the primary focus due to its requirement in processing the bulk of nsps found encoded in the viral genome. Two Mpro inhibitors are now available for the treatment of COVID-19: nirmatrelvir [41] and ensitrelvir [42]. Preclinical candidates targeting PLpro are now emerging (e.g. [43,44]), and these are likely to complement Mpro inhibitors because of the dual role of PLpro in nsp processing and removal of ubiquitin and Interferon-Stimulated Gene 15 (ISG15) from viral and host proteins, the latter of which may have consequences for immune cell signalling and disease severity [45]. DMS screens have been performed on both proteases to understand their sequence–function relationships and elucidate drug escape mechanisms for emerging therapeutics [14–16,46].

### Mpro

Mpro, or nsp5, is a cysteine protease that releases 11 of 16 viral nsps from ORF1a and 1ab, including itself. The Mpro cleavage sites are conserved and characterised by a glutamine residue at the C-terminal to the cleavage site [47]. Mpro forms an obligate dimer that is crucial for its function, with the N-terminus of each monomer contributing to the positioning of the catalytic dyad of the other monomer’s active site [48] (Figure 1b(**ii**)). Disruption of dimerisation can significantly reduce its proteolytic activity, making the dimer interface a promising target for antiviral drug development [49].

Two groups have published DMS screens of Mpro using yeast as a model system [14,15], each with >99% coverage of single-site substitutions. Mpro is toxic when overexpressed, causing growth retardation likely due to the cleavage of essential yeast proteins. This property allows Mpro activity to be measured based on the enrichment of inactive variants over time (Figure 3b). The Bolon laboratory [14] additionally used two orthogonal screens with similar results: a Förster resonance energy transfer (FRET)-based biosensor composed of cyan fluorescent protein and yellow fluorescent protein separated by a short Mpro-cleavable sequence, and a split transcription factor screen where cleavage of a biosensor reduced the expression of GFP (Figure 3c). All three assays and both papers yielded similar results identifying residues buried in the globular structure and dimerisation interface, residues in and around the active site, and a putative network of residues that provide an allosteric pathway between the active site and the dimerisation interface. One limitation of both of these studies was that Mpro abundance was not measured, so it remains unclear whether variants cause functional or structural defects, and this complicates mechanistic interpretation. Nevertheless, the sequence–function maps generated in these studies allow drug contact sites to be assessed for their mutability and thereby predict pathways for viral drug escape.

In a follow-up paper [46], the Bolon laboratory assessed Mpro resistance to nirmatrelvir and ensitrelvir by DMS using their yeast cell growth assay. These were challenging experiments, as the permeability of yeast to small-molecule compounds is limited and yeast possess numerous drug efflux pumps. To work around these issues, the screen was performed using a yeast strain with four efflux pumps knocked out, supplementing media with a low concentration of SDS and using both drugs at concentrations 100-fold over what is used to effect viral inhibition in mammalian systems. These complications notwithstanding, drug resistance scores could be calculated that showed mutations at key drug contacts ablated inhibition, and a subset of the observed mutations has been reported elsewhere, suggesting the results of this screen are reasonable [50].

When drug resistance mutations were assessed *en masse,* two important features emerged. Residues outside the substrate-binding envelope (E) were more susceptible to resistance-causing mutations than positions inside the E, and there was a trend towards these mutations causing reduced Mpro fitness. The latter feature provides a selective pressure to stabilise Mpro, leading to the accumulation of multiple mutations that both evade therapeutics and restore fitness. For instance, the combination of E166V and L50F has been measured as spontaneously arising in live virus passaged in the presence of nirmatrelvir [51]. E166V was shown in the DMS screen to both reduce Mpro activity and provide resistance to both nirmatrelvir and ensitrelvir, while L50F enhanced Mpro activity. Thus, the DMS studies, while typically only measuring single-site substitutions, can still be mechanistically informative in predicting the additive consequences of independent mutations. To date, none of the variants in this study have been observed at appreciable frequency in circulating viruses, likely because access to these medications is limited to high-risk patients in most countries. This DMS study additionally identified some drug-resistance mutations that impaired both currently available drugs, implying that viral evolution could render the entire therapeutic arsenal against Mpro ineffective with only a single mutation. The potential for this scenario argues for further drug development in this space.

### PLpro

PLpro is a domain of nsp3 (Figure 1b(**i**)), which is the longest nsp in the SARS-CoV-2 genome. A recent study utilising cryo-electron tomography revealed how nsp3 together with nsp4 forms an RNA translocating pore that spans double-membrane vesicles, a compartment for viral RNA synthesis and replication [2]. Although PLpro plays a structural role in the crown of the nsp3-nsp4 pore, it also plays an essential enzymatic function in releasing nsp1 and nsp2 from the polyproteins to enable viral replication. It also deconjugates ubiquitin and ISG15 protein modifications, functions that are thought to moderate cellular innate immunity and favour virus expansion [45,52]. These enzymatic activities of PLpro remain intact even when PLpro is expressed in isolation from the full nsp3 protein. Therefore, unlike Mpro, inhibiting PLpro may both prevent the self-processing of viral polyprotein chains and interfere with the ability of the virus to modulate immune response. Our lab performed DMS on PLpro to understand its biology and potential liabilities as a drug target [16].

While enforced expression of Mpro inhibits cell growth, PLpro does not. Instead, we developed a FRET biosensor containing donor mClover3 attached to acceptor mRuby3 via a short linker that contained a sequence susceptible to PLpro cleavage (Figure 3c) [16]. The status of the biosensor could be monitored by flow cytometry, and FRET was lost upon PLpro expression in mammalian cells. This facile readout enabled drug-escape data to be collected at concentrations closer to those that are biologically relevant without the need for special cell lines or SDS to permeabilise yeast cell walls, as in the prior Mpro drug-escape study [46].

Performed in HEK293T cells with PLpro retrovirally introduced under doxycycline control, our assay monitored biosensor cleavage and calculated activity scores for almost all of the 6300 possible PLpro single-amino-acid substitutions (99.4%). We coupled this to a similar screen that expressed PLpro variants fused to mClover3 to monitor PLpro abundance, allowing us to discriminate inactive PLpro variants that were stably expressed in the cell from PLpro variants that were misfolded and rapidly degraded. This facilitated the identification of a network of residues that extends from the active site of the protein towards the site that engages ubiquitin and ISG15, suggesting a previously unappreciated allosteric network that governs catalytic activity.

The active site of PLpro is shallow and not amenable to targeting directly with therapeutic molecules. Instead, drug development has focussed on the deep substrate pocket (S4) that engages a large hydrophobic residue (typically leucine) at the P4 position of the cleavage site. We were able to test some lead compounds with moderate activity for resistance mutations, confirming that our DMS experiments had the sensitivity to identify such mutations and revealing that the non-conserved blocking loop lining the S4 pocket is a liability for drug development. These experiments also identified M208 as a modulator of flexibility in the S4 pocket, to which all reported lead compounds bind.

While PLpro inhibitors are still in early development, the above DMS study has provided a comprehensive map of PLpro functional substitutions. This has defined the landscape of variants open to the virus, should selective pressure be applied by the availability of an effective inhibitor. It also highlights the challenges that makers of therapeutics must overcome to ensure continued efficacy, including the structural flexibility of the S4 pocket and its susceptibility to resistance mutations.

## Nucleocapsid

Throughout the pandemic, rapid antigen testing has been used to quickly identify infected individuals, allowing them to be isolated to limit viral spread. These tests, from a variety of manufacturers, use different mAbs to capture and detect the N protein (Figure 1b(**iv**)) that condenses the viral RNA genome. Mutations in the N protein that prevent antibody binding would render a false-negative test result, providing a transmission advantage in settings of heavy testing. DMS using a surface displayed N on mammalian cells (99.5% coverage of possible single-site substitutions) (Figure 3a) has been used to pre-emptively determine the possible mutations that would allow escape from a variety of antibodies used in commercial test kits [17]. The results showed the binding epitopes of many antibodies had commonalities that could affect multiple test kits. Coupling DMS with surveillance efforts going forward would thus ensure testing remains accurate, enhancing public health efforts to prevent unchecked viral spread.

## Current limitations and future opportunities

DMS has provided insights into the selective pressures driving the evolution of the spike protein and laid the groundwork for surveillance of variants that may arise in response to immune pressure and emerging therapeutics. Indeed, DMS datasets have shown that antibodies targeting different epitopes on the SARS-CoV-2 RBD exhibit predictable capacities to cross-neutralise emerging strains with mutations at these sites. Leveraging these DMS datasets, identification of previously circulating variants in the community, and the extent of viral spread determined by wastewater surveillance, researchers developed a model to forecast the competitive dynamics between strains from a temporal snapshot of circulating variants [53]. Tested on retrospective data, this model successfully predicted which variants at a given timepoint would become dominant and forecasted the timing and magnitude of subsequent COVID-19 waves. This approach could potentially inform rapid vaccine deployment targeted to specific geographical areas, addressing a critical need in the absence of predictable SARS-CoV-2 seasonality, which currently complicates the design of annual vaccination strategies.

While the aforementioned example highlights the power of rich datasets, current DMS assays and targets are predominantly limited to assessing narrow aspects of SARS-CoV-2 biology. Antibody responses to RBD have been well characterised, but other spike domains are less well defined. It is clear that T-cell immunity plays a role in protection against disease, but T-cell epitopes that are presented in the context of the major histocompatibility complex are difficult to assess with assays amenable to DMS. The development of replicon systems, which contain the necessary sequences for replication of a minimal genome within a cell but not viral packaging [54,55], provides a basis for assessing the contribution of many more nsps to viral replication. These could be used, for example, to scan nsp12, which is the target of molnupiravir and remdesivir, and assess the criticality of other less well-characterised proteins in the SARS-CoV-2 genome.

DMS is increasingly valuable not only as a tool for interrogating protein function but also as a source of critical benchmarking data for developing and refining Artificial Intelligence and Machine Learning-based models, e.g. [56–58]. As computational frameworks continue to evolve, DMS datasets can help ground model predictions in experimental reality and inform model calibration and benchmarking.

DMS is still a relatively new technique, but it has contributed significantly to our understanding of this newly emerged pathogen. Importantly, the approaches exemplified in this review can be applied to any emerging pathogen and are indeed improving our understanding of existing pathogens such as influenza [59], respiratory syncytial virus [60], HIV [61] and Nipah virus [62], to name but a few. Improvements in assay technologies, cloning strategies and sequencing throughput will only enhance its capabilities.

**Perspectives**

- Deep mutational scanning (DMS) has been instrumental in mapping viral protein function and evolution, providing critical insights into immune escape and the potential for drug resistance during the severe acquired respiratory syndrome-coronavirus-2 (SARS-CoV-2) pandemic. This approach enables rapid assessment of mutation impacts, guiding vaccine updates and therapeutic development.
- Current research has leveraged DMS to systematically assess the functional and structural consequences of mutations in key SARS-CoV-2 proteins, such as spike, main protease, papain-like protease and nucleocapsid. These studies have highlighted conserved vulnerabilities and adaptive strategies of the virus.
- Expanding DMS to a broader range of viral proteins and host factors, while developing new assays to measure variant function, will provide a more complete picture of virus–host interactions, helping to refine antiviral strategies.

## Abbreviations

ACE2: Angiotensin-converting enzyme 2
COVID-19: Coronavirus disease 2019
DMS: deep mutational scanning
E: envelope
FRET: Förster resonance energy transfer
GFP: green fluorescent protein
HCV: hepatitis C virus
HEK293T: human embryonic kidney 293T
HIV-1: human immunodeficiency virus subtype 1
ISG15: Interferon-stimulated gene 15
M: membrane
Mpro: main protease
N: nucleocapsid
NTD: N-terminal domain
ORF: Open reading frame
PLpro: papain-like protease
RBD: receptor-binding domain
SARS-CoV-2: severe acquired respiratory syndrome-coronavirus 2
mAb: monoclonal antibody
nsp(s): non-structural protein(s)

## References

[1] Zhu N. Zhang D. Wang W. Li X. Yang B. Song J. et al 2020 A novel coronavirus from patients with pneumonia in China, 2019 N. Engl. J. Med. 382 727 733 10.1056/NEJMoa2001017 31978945 PMC7092803

[2] Huang Y. Wang T. Zhong L. Zhang W. Zhang Y. Yu X. et al 2024 Molecular architecture of coronavirus double-membrane vesicle pore complex Nature 633 224 231 10.1038/s41586-024-07817-y 39143215 PMC11374677

[3] Zhang L. Lin D. Sun X. Curth U. Drosten C. Sauerhering L. et al 2020 Crystal structure of SARS-CoV-2 main protease provides a basis for design of improved α-ketoamide inhibitors Science 368 409 412 10.1126/science.abb3405 32198291 PMC7164518

[4] Wrapp D. Wang N. Corbett K.S. Goldsmith J.A. Hsieh C.-L. Abiona O. et al 2020 Cryo-EM structure of the 2019-nCoV spike in the prefusion conformation Science 367 1260 1263 10.1126/science.abb2507 32075877 PMC7164637

[5] Shi W. Cai Y. Zhu H. Peng H. Voyer J. Rits-Volloch S. et al 2023 Cryo-EM structure of SARS-CoV-2 postfusion spike in membrane Nature 619 403 409 10.1038/s41586-023-06273-4 37285872

[6] Abramson J. Adler J. Dunger J. Evans R. Green T. Pritzel A. et al 2024 Accurate structure prediction of biomolecular interactions with AlphaFold 3 Nature 630 493 500 10.1038/s41586-024-07487-w 38718835 PMC11168924

[7] Haque A. Pant AB 2024 The coevolution of Covid-19 and host immunity Exploration of Medicine 5 167 184 10.37349/emed.2024.00214

[8] Starr T.N. Greaney A.J. Hilton S.K. Ellis D. Crawford K.H.D. Dingens A.S et al 2020 Deep Mutational Scanning of SARS-CoV-2 receptor binding domain reveals constraints on folding and ACE2 Binding Cell 182 1295 1310 10.1016/j.cell.2020.08.012 32841599 PMC7418704

[9] Ouyang W.O. Tan T.J.C. Lei R. Song G. Kieffer C. Andrabi R. et al 2022 Probing the biophysical constraints of SARS-CoV-2 spike N-terminal domain using deep mutational scanning Sci. Adv. 8 eadd7221 10.1126/sciadv.add7221 36417523 PMC9683733

[10] Kim Y.C. Dema B. Reyes-Sandoval A 2020 COVID-19 vaccines: breaking record times to first-in-human trials NPJ Vaccines 5 34 10.1038/s41541-020-0188-3 32377399 PMC7193619

[11] Fowler D.M. Fields S 2014 Deep mutational scanning: a new style of protein science Nat. Methods 11 801 807 10.1038/nmeth.3027 25075907 PMC4410700

[12] Hietpas R.T. Jensen J.D. Bolon D.N.A 2011 Experimental illumination of a fitness landscape Proc. Natl. Acad. Sci. U.S.A. 108 7896 7901 10.1073/pnas.1016024108 21464309 PMC3093508

[13] Araya C.L. Fowler DM 2011 Deep mutational scanning: assessing protein function on a massive scale Trends Biotechnol. 29 435 442 10.1016/j.tibtech.2011.04.003 21561674 PMC3159719

[14] Flynn J.M. Samant N. Schneider-Nachum G. Barkan D.T. Yilmaz N.K. Schiffer C.A. et al 2022 Comprehensive fitness landscape of SARS-CoV-2 M^pro^ reveals insights into viral resistance mechanisms Elife 11 e77433 10.7554/eLife.77433 35723575 PMC9323007

[15] Iketani S. Hong S.J. Sheng J. Bahari F. Culbertson B. Atanaki F.F et al 2022 Functional map of SARS-CoV-2 3CL protease reveals tolerant and immutable sites Cell Host Microbe 30 1354 1362 10.1016/j.chom.2022.08.003 36029764 PMC9365866

[16] Wu X. Go M. Nguyen J.V. Kuchel N.W. Lu B.G.C. Zeglinski K. et al 2024 Mutational profiling of SARS-CoV-2 papain-like protease reveals requirements for function, structure, and drug escape Nat. Commun. 15 6219 10.1038/s41467-024-50566-9 39043718 PMC11266423

[17] Frank F. Keen M.M. Rao A. Bassit L. Liu X. Bowers H.B et al 2022 Deep mutational scanning identifies SARS-CoV-2 Nucleocapsid escape mutations of currently available rapid antigen tests Cell 185 3603 3616 10.1016/j.cell.2022.08.010 36084631 PMC9420710

[18] Dadonaite B. Brown J. McMahon T.E. Farrell A.G. Figgins M.D. Asarnow D. et al 2024 Spike deep mutational scanning helps predict success of SARS-CoV-2 clades Nature 631 617 626 10.1038/s41586-024-07636-1 38961298 PMC11254757

[19] Dadonaite B. Crawford K.H.D. Radford C.E. Farrell A.G. Yu T.C. Hannon W.W et al 2023 A pseudovirus system enables deep mutational scanning of the full SARS-CoV-2 spike Cell 186 1263 1278 10.1016/j.cell.2023.02.001 36868218 PMC9922669

[20] Kugathasan R. Sukhova K. Moshe M. Kellam P. Barclay W 2023 Deep mutagenesis scanning using whole trimeric SARS-CoV-2 spike highlights the importance of NTD-RBD interactions in determining spike phenotype PLoS Pathog. 19 e1011545 10.1371/journal.ppat.1011545 37535672 PMC10426949

[21] Lei R. Qing E. Odle A. Yuan M. Gunawardene C.D. Tan T.J.C. et al 2024 Functional and antigenic characterization of SARS-CoV-2 spike fusion peptide by deep mutational scanning Nat. Commun. 15 4056 10.1038/s41467-024-48104-8 38744813 PMC11094058

[22] Hadfield J. Megill C. Bell S.M. Huddleston J. Potter B. Callender C. et al 2018 Nextstrain: real-time tracking of pathogen evolution Bioinformatics 34 4121 4123 10.1093/bioinformatics/bty407 29790939 PMC6247931

[23] Ren W. Lan J. Ju X. Gong M. Long Q. Zhu Z. et al 2021 Mutation Y453F in the spike protein of SARS-CoV-2 enhances interaction with the mink ACE2 receptor for host adaption PLoS Pathog. 17 e1010053 10.1371/journal.ppat.1010053 34748603 PMC8601601

[24] Bate N. Savva C.G. Moody P.C.E. Brown E.A. Evans S.E. Ball J.K. et al 2022 In vitro evolution predicts emerging SARS-CoV-2 mutations with high affinity for ACE2 and cross-species binding PLoS Pathog. 18 e1010733 10.1371/journal.ppat.1010733 35849637 PMC9333441

[25] Starr T.N. Greaney A.J. Hannon W.W. Loes A.N. Hauser K. Dillen J.R. et al 2022 Shifting mutational constraints in the SARS-CoV-2 receptor-binding domain during viral evolution Science 377 420 424 eabo7896 10.1126/science.abo7896 35762884 PMC9273037

[26] Starr T.N. Greaney A.J. Stewart C.M. Walls A.C. Hannon W.W. Veesler D. et al 2022 Deep mutational scans for ACE2 binding, RBD expression, and antibody escape in the SARS-CoV-2 Omicron BA.1 and BA.2 receptor-binding domains PLoS Pathog. 18 e1010951 10.1371/journal.ppat.1010951 36399443 PMC9674177

[27] Greaney A.J. Starr T.N. Gilchuk P. Zost S.J. Binshtein E. Loes A.N. et al Complete mapping of mutations to the SARS-CoV-2 spike receptor-binding domain that escape antibody recognition Microbiology 10.1101/2020.09.10.292078 PMC7676316 33259788

[28] Loes A.N. Crawford K.H.D. Starr T.N. Malone K.D. Chu H.Y et al 2021 Comprehensive mapping of mutations in the SARS-CoV-2 receptor-binding domain that affect recognition by polyclonal human plasma antibodies Cell Host Microbe 29 463 476 10.1016/j.chom.2021.02.003 33592168 PMC7869748

[29] Greaney A.J. Starr T.N. Barnes C.O. Weisblum Y. Schmidt F. Caskey M. et al 2021 a) Mapping mutations to the SARS-CoV-2 RBD that escape binding by different classes of antibodies Nat. Commun. 12 4196 10.1038/s41467-021-24435-8 34234131 PMC8263750

[30] Greaney A.J. Starr T.N. Eguia R.T. Loes A.N. Khan K. Karim F. et al 2022 A SARS-CoV-2 variant elicits an antibody response with a shifted immunodominance hierarchy PLoS Pathog. 18 e1010248 10.1371/journal.ppat.1010248 35134084 PMC8856557

[31] Greaney A.J. Loes A.N. Gentles L.E. Crawford K.H.D. Starr T.N. Malone K.D. et al 2021 Antibodies elicited by mRNA-1273 vaccination bind more broadly to the receptor binding domain than do those from SARS-CoV-2 infection Sci. Transl. Med. 13 eabi9915 10.1126/scitranslmed.abi9915 34103407 PMC8369496

[32] Cohen A.A. Van Doremalen N. Greaney A.J. Andersen H. Sharma A. Starr T.N et al 2022 Mosaic RBD nanoparticles protect against challenge by diverse sarbecoviruses in animal models Science 377 eabq0839 10.1126/science.abq0839 35857620 PMC9273039

[33] Voss W.N. Hou Y.J. Johnson N.V. Delidakis G. Kim J.E. Javanmardi K. et al 2021 Prevalent, protective, and convergent IgG recognition of SARS-CoV-2 non-RBD spike epitopes Science 372 1108 1112 eabg5268 10.1126/science.abg5268 33947773 PMC8224265

[34] Unione L. Moure M.J. Lenza M.P. Oyenarte I. Ereño-Orbea J. Ardá A. et al 2022 The SARS-CoV-2 Spike glycoprotein directly binds exogeneous sialic acids: a NMR View Angew. Chem. Int. Ed. Engl. 61 e202201432 10.1002/anie.202201432 35191576 PMC9074024

[35] Li C.J. Chang SC 2023 SARS-CoV-2 spike S2-specific neutralizing antibodies Emerg. Microbes Infect. 12 2220582 10.1080/22221751.2023.2220582 37254830 PMC10274517

[36] Pallesen J. Wang N. Corbett K.S. Wrapp D. Kirchdoerfer R.N. Turner H.L. et al 2017 Immunogenicity and structures of a rationally designed prefusion MERS-CoV spike antigen Proc. Natl. Acad. Sci. U.S.A. 114 E7348 E7357 10.1073/pnas.1707304114 28807998 PMC5584442

[37] Tan T.J.C. Mou Z. Lei R. Ouyang W.O. Yuan M. Song G. et al 2023 High-throughput identification of prefusion-stabilizing mutations in SARS-CoV-2 spike Nat. Commun. 14 2003 10.1038/s41467-023-37786-1 37037866 PMC10086000

[38] Rubio A.A. Baharani V.A. Dadonaite B. Parada M. Abernathy M.E. Wang Z. et al 2024 Bispecific antibodies with broad neutralization potency against SARS-CoV-2 variants of concern bioRxiv 2024.05.05.592584 10.1101/2024.05.05.592584 38766244 PMC11100608

[39] Sheward D.J. Pushparaj P. Das H. Greaney A.J. Kim C. Kim S. et al 2024 Structural basis of broad SARS-CoV-2 cross-neutralization by affinity-matured public antibodies Cell Rep. Med. 5 101577 10.1016/j.xcrm.2024.101577 38761799 PMC11228396

[40] Majerová T. Konvalinka J 2022 Viral proteases as therapeutic targets Mol. Aspects Med 88 101159 10.1016/j.mam.2022.101159 36459838 PMC9706241

[41] Owen D.R. Allerton C.M.N. Anderson A.S. Aschenbrenner L. Avery M. Berritt S et al 2021 An oral SARS-CoV-2 ^M^ Science 374 1586 1593 10.1126/science.abl4784 34726479

[42] Unoh Y. Uehara S. Nakahara K. Nobori H. Yamatsu Y. Yamamoto S. et al 2022 Discovery of S-217622, a Noncovalent Oral SARS-CoV-2 3CL Protease Inhibitor Clinical Candidate for Treating COVID-19 J. Med. Chem. 65 6499 6512 10.1021/acs.jmedchem.2c00117 35352927 PMC8982737

[43] Garnsey M.R. Robinson M.C. Nguyen L.T. Cardin R. Tillotson J. Mashalidis E. et al 2024 Discovery of SARS-CoV-2 papain-like protease (PL^pro^) inhibitors with efficacy in a murine infection model Sci. Adv. 10 eado4288 10.1126/sciadv.ado4288 39213347 PMC11364104

[44] Tan B. Zhang X. Ansari A. Jadhav P. Tan H. Li K. et al 2024 Design of a SARS-CoV-2 papain-like protease inhibitor with antiviral efficacy in a mouse model Science 383 1434 1440 10.1126/science.adm9724 38547259 PMC12178660

[45] Gold I.M. Reis N. Glaser F. Glickman M.H 2022 Coronaviral PLpro proteases and the immunomodulatory roles of conjugated versus free Interferon Stimulated Gene product-15 (ISG15) Semin. Cell Dev. Biol 132 16 26 10.1016/j.semcdb.2022.06.005 35764457 PMC9233553

[46] Flynn J.M. Huang Q.Y.J. Zvornicanin S.N. Schneider-Nachum G. Shaqra A.M. Yilmaz N.K. et al 2023 Systematic analyses of the resistance potential of drugs targeting SARS-CoV-2 Main Protease ACS Infect. Dis. 9 1372 1386 10.1021/acsinfecdis.3c00125 37390404 PMC11161032

[47] Hegyi A. Ziebuhr J 2002 Conservation of substrate specificities among coronavirus main proteases J. Gen. Virol. 83 595 599 10.1099/0022-1317-83-3-595 11842254

[48] Jin Z. Du X. Xu Y. Deng Y. Liu M. Zhao Y. et al 2020 Structure of Mpro from SARS-CoV-2 and discovery of its inhibitors Nature 582 289 293 10.1038/s41586-020-2223-y 32272481

[49] Goyal B. Goyal D 2020 Targeting the dimerization of the main protease of coronaviruses: a potential broad-spectrum therapeutic strategy ACS Comb. Sci. 22 297 305 10.1021/acscombsci.0c00058 32402186

[50] Iketani S. Mohri H. Culbertson B. Hong S.J. Duan Y. Luck M.I. et al 2023 Multiple pathways for SARS-CoV-2 resistance to nirmatrelvir Nature 613 558 564 10.1038/s41586-022-05514-2 36351451 PMC9849135

[51] Zhou Y. Gammeltoft K.A. Ryberg L.A. Pham L.V. Tjørnelund H.D. Binderup A. et al 2022 Nirmatrelvir-resistant SARS-CoV-2 variants with high fitness in an infectious cell culture system Sci. Adv. 8 10.1126/sciadv.add7197 PMC9770952 36542720

[52] Rhamadianti A.F. Abe T. Tanaka T. Ono C. Katayama H. Makino Y et al 2024 SARS-CoV-2 papain-like protease inhibits ISGylation of the viral nucleocapsid protein to evade host anti-viral immunity J. Virol 98 e0085524 10.1128/jvi.00855-24 39120134 PMC11406913

[53] Raharinirina N.A. Gubela N. Börnigen D. Smith M.R. Oh D.-Y. Budt M. et al 2025 SARS-CoV-2 evolution on a dynamic immune landscape Nature 639 196 204 10.1038/s41586-024-08477-8 39880955 PMC11882442

[54] He X. Quan S. Xu M. Rodriguez S. Goh S.L. Wei J. et al 2021 Generation of SARS-CoV-2 reporter replicon for high-throughput antiviral screening and testing Proc. Natl. Acad. Sci. U.S.A 118 10.1073/pnas.2025866118 PMC8053989 33766889

[55] Tanaka T. Saito A. Suzuki T. Miyamoto Y. Takayama K. Okamoto T et al 2022 Establishment of a stable SARS-CoV-2 replicon system for application in high-throughput screening Antiviral Res 199 105268 10.1016/j.antiviral.2022.105268 35271914 PMC8900913

[56] Luo Y. Jiang G. Yu T. Liu Y. Vo L. Ding H. et al 2021 ECNet is an evolutionary context-integrated deep learning framework for protein engineering Nat. Commun. 12 5743 10.1038/s41467-021-25976-8 34593817 PMC8484459

[57] Frazer J. Notin P. Dias M. Gomez A. Min J.K. Brock K. et al 2021 Disease variant prediction with deep generative models of evolutionary data Nature 599 91 95 10.1038/s41586-021-04043-8 34707284

[58] Cheng J. Novati G. Pan J. Bycroft C. Žemgulytė A. Applebaum T. et al 2023 Accurate proteome-wide missense variant effect prediction with AlphaMissense Science 381 eadg7492 10.1126/science.adg7492 37733863

[59] Dadonaite B. Ahn J.J. Ort J.T. Yu J. Furey C. Dosey A. et al 2024 Deep mutational scanning of H5 hemagglutinin to inform influenza virus surveillance PLoS Biol. 22 e3002916 10.1371/journal.pbio.3002916 39531474 PMC11584116

[60] Simonich C.A.L. McMahon T.E. Ju X. Yu T.C. Brunette N. Stevens-Ayers T et al 2025 RSV F evolution escapes some monoclonal antibodies but does not strongly erode neutralization by human polyclonal sera J. Virol 99 e0053125 10.1128/jvi.00531-25 40607811 PMC12282093

[61] Kim S. Radford C.E. Xu D. Zhong J. Do J. Pham D.M. et al 2025 A broad antibody with enhanced HIV-1 neutralization via bispecific antibody-mediated prepositioning Nat. Commun. 16 4617 10.1038/s41467-025-60035-6 40383778 PMC12086220

[62] Larsen B.B. McMahon T. Brown J.T. Wang Z. Radford C.E. Crowe J.E. Jr et al 2025 Functional and antigenic landscape of the Nipah virus receptor-binding protein Cell 188 2480 2494 10.1016/j.cell.2025.02.030 40132580 PMC12048240

